# Knowledge of lactation amenorrhea method among postpartum women in Ethiopia: a facility-based cross-sectional study

**DOI:** 10.1038/s41598-023-42196-w

**Published:** 2023-09-09

**Authors:** Tadesse Gure Eticha, Sagni Girma, Galana Mamo, Fekede Asefa, Abdi Birhanu, Bedasa Taye, Addisu Alemu, Kabtamu Niguse, Abel Gedefaw, Tinsae Genet, Demesew Amenu, Thomas Mekuria, Abera Kenay Tura

**Affiliations:** 1 Department of Obstetrics and Gynecology, School of Medicine, College of Health and Medical Sciences, Haramaya University, Harar, Ethiopia; 2 School of Nursing and Midwifery, College of Health and Medical Sciences, Haramaya University, Harar, Ethiopia; 3 Department of Obstetrics and Gynaecology, Leiden University Medical Centre, Leiden, The Netherlands; 4 School of Public Health, College of Health and Medical Sciences, Haramaya University, Harar, Ethiopia; 5 Department of Pediatrics, College of Medicine, The University of Tennessee Health Science Center (UTHSC)-Oak Ridge National Laboratory (ORNL) Center for Biomedical Informatics, Memphis, TN38103 USA; 6 School of Medicine, College of Health and Medical Sciences, Haramaya University, Harar, Ethiopia; 7 School of Medicine, College of Health and Medical Sciences, Hawassa University, Hawassa, Ethiopia; 8 School of Medicine, College of Health and Medical Sciences, University of Gondar, Gondar, Ethiopia; 9 School of Medicine, College of Health and Medical Sciences, Jimma University, Jimma, Ethiopia; 10 School of Medicine, St. Paul’s Millennium Medical College Hospital, Addis Ababa, Ethiopia

**Keywords:** Health care, Medical research

## Abstract

While the importance of knowledge about contraceptives in improving their utilization and thereby reducing the risk of unintended pregnancies is well documented, there are limited studies documented about the Lactational Amenorrhea Method (LAM). Thus, understanding the knowledge of postpartum mothers about LAM is essential for designing tailored interventions. This study assessed the level of knowledge about LAM and its associated factors among postpartum mothers in Ethiopia. A facility-based cross-sectional study was conducted among 3148 randomly selected postpartum participants. The study utilized multistage sampling approach in hospitals located across five regions and one city administration in Ethiopia. Data were collected using face-to-face interviews at discharge. A participant was categorized as having knowledge of LAM if she correctly answered the three LAM criteria: amenorrhea, the first 6 months, and exclusive breast feeding. A binary logistic regression model was used to identify factors associated with knowledge of LAM. Variables with *p* < 0.25 in the binary logistic regression were included in the multiple logistic regression. Then, associations were described using the adjusted odds ratio (AOR) along with the 95% confidence interval (CI), and statistical significance was declared at *p* < 0.05. Only four in 10 participants (40.6%; 95% CI 38.9–42.3) had knowledge of LAM. Participants who attended college or above educational level (AOR = 2.1, 95% CI 1.5–2.8), those with parity of two (AOR = 2.3; 95% CI 1.6–3.6) or more than two (AOR = 2.4; 95% CI 1.5–4.0), those who expressed a desire for further fertility (AOR = 1.3; 95% CI 1.1–1.5), individuals who received counselling on LAM (AOR = 3.0; 95% CI 2.6–3.7), and those who gave birth in hospital (AOR = 2.6; 95% CI 1.4–2.6) had higher odds of knowledge about LAM, compared to their counter parts. In contrary, participants resided far away from health facilities had 30% lower odd of knowledge about LAM compared to those resided near the health facilities (AOR = 0.70; 95% CI 0.6–0.8). The proportion of participants who had knowledge of LAM was low. Strengthening counseling about LAM during antenatal care and delivery with due attention to women with limited access to health facilities should be considered for increasing their level of knowledge on LAM.

## Introduction

The Lactational Amenorrhea Method (LAM) is a natural defense mechanism against pregnancy triggered by breastfeeding^1^. It is a natural contraceptive method that is highly safe, effective, inexpensive, available, and accessible to many mothers in the first 6 months postpartum^2–4^. The LAM provides 98% protection against pregnancy if the three Bellagio Consensus (LAM) criteria are met: (1) the menses have not returned, (2) the baby is fully or nearly fully breastfed, and (3) the baby is less than 6 months old^3,5,6^. As with any other contraceptive method, the efficacy of LAM is affected by its correct use (fulfillment of three LAM criteria), which might be affected by knowledge about breastfeeding and its role in the temporary suppression of fertility^7–9^.

Poor knowledge about LAM and its related criteria as a contraceptive method decreases its efficacy among postpartum mothers, which might result in pregnancy^4^. Given that 90% of mothers worldwide want to delay or limit their subsequent pregnancies during the postpartum period^10^, using LAM can prevent unwanted pregnancies^11^. Although the efficacy of LAM as a method of contraception cannot be questioned, its reliability is affected by the knowledge of users about when to use it as a contraceptive. According to the literature on the efficacy of LAM, efficacy ranges from 98 to 100% in correctly used participants in the first 6 months of the postpartum period. Pregnancy rates were higher among those who did not meet the LAM criteria^12–14^. Despite high unmet demand for family planning at the national level (i.e., 80%)^15–17^, only 0.1% of mothers rely on LAM as a contraceptive, according to the EDHS 2016 in Ethiopia^18^.

Despite the many benefits of breastfeeding (contraceptive method, providing good nutrition for infants, ease of use, and availability^19,20^, knowledge of LAM is less emphasized in the Ethiopian Health Sector Transformation Plan (HSTP) agenda^15,21^. Given the dearth of literature on the knowledge of LAM in Ethiopia, a country with a high fertility rate^18^, understanding mother’s knowledge and associated factors about LAM is essential. In this study, we assessed knowledge of LAM and its associated factors among postpartum mothers in Ethiopia.

## Methods

### Study setting and design

This study is part of a larger multi-center national study on the efficacy of LAM in Ethiopia, which was conducted in selected major public referral hospitals in Amhara, Oromia, the Southern Nations and Nationalities People Region, Harari, Somali regions, and Addis Ababa City Administration. The hospitals were affiliated with major medical schools (Hiwot Fana Comprehensive Specialized University Hospital, Karamara Hospital, Hawassa Referral Hospital, Jimma University Hospital, Gondar Comprehensive Specialized University Hospital, and St. Paul’s Millennium Medical College Hospital). Moreover, five health centers directly referring to respective hospitals were included. The study was conducted from March 1, 2017 to December 31, 2018.

### Population and sample

**Figure 1:**
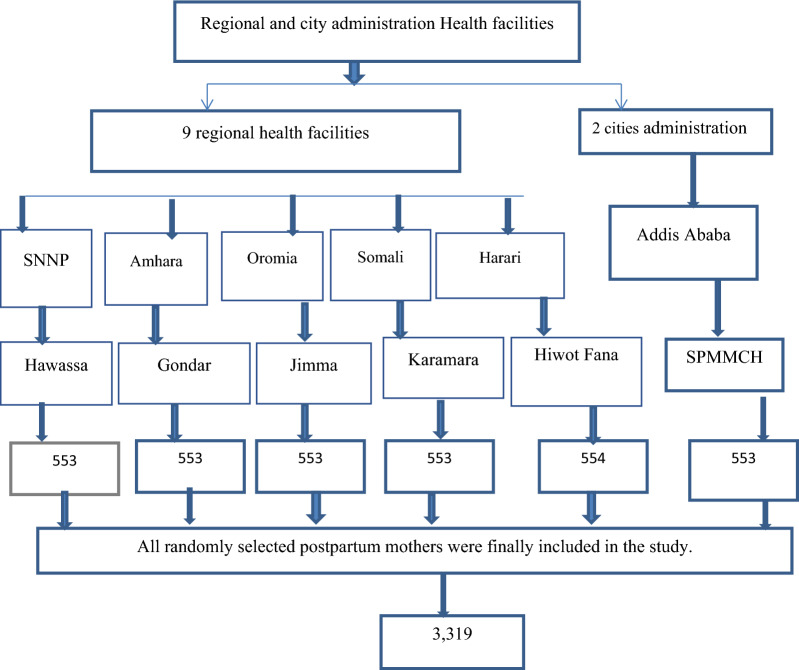
Diagrammatic presentation of sampling procedure for study participants’ selection in Ethiopia, 2018. *SNNP* Southern Nations and Nationalities People Region, *SPMMCH* St. Paul’s Millennium Medical College Hospital.

All postpartum mothers in Ethiopia during study period were our source population while mothers who gave birth in the participating hospitals and health centers during data collection time were considered the study population, while those who participated in the study were study subjects. The sample size for the study was calculated with the following assumptions: 95% confidence interval, Z = 1.96, margin of error (d) of 2%, proportion 26%^22^, 20% non-response, and design effect of 1.5. The final sample size was determined to be 3319. From the nine national regional states and two city administrations (during the study period), five regional states and one city administration were randomly selected. In each selected region and city administration, one referral hospital and five nearest health centers were included in the study. The calculated samples were proportionally allocated to the selected health facilities as described in Fig. 1.

### Variables and measurements

An outcome variable of this study was knowledge about LAM. Sociodemographic conditions (age, religion, occupation, residence, educational status, marital status, partner’s education, and occupation, obstetric-related information, sexual status, and information about LAM were considered independent variables. A participant was considered knowledgeable and categorized as ‘yes’ if she correctly identified the three LAM criteria—amenorrhea, infant’s age less than 6 months, and achieving exclusive breast feeding—or ‘no’ otherwise^9^.

### Data collection procedure and quality control

A standard structured questionnaire adapted from the 2016 Ethiopian demographic and health survey questionnaire^18^ was used to collect information through interviews from participants at discharge. The tool was translated into local languages (Afan Oromo, Amharic, and Af-Somali) and administered by trained female research assistants fluent in the local languages. The data were collected using ODK Collect. A pretest was conducted among 5% of the sample population in the hospital that was not included in the study prior to the actual data collection. Adequacy of the checklist was evaluated and ambiguous questions were modified. Additionally, daily checks were made by the respective site supervisors to ensure its accuracy and consistency.

### Data processing and analysis

Data from the ODK collection was exported to MS Excel. STATA 14 was used for further data cleaning and analysis. The data were summarized using tables, figures, and texts. Frequency and percentages were used for categorical variables, and the mean with standard deviation was used to summarize the continuous variables. On top of the modeling, key model assumptions, including chi-square, Multicollinearity, and linearity in the Logit for continuous variables of logistic regression, were assessed. Bi-variable binary logistic models were fitted for each predictor. Predictors from the bi-variable analysis that had a *p* value of less than 0.25 were included in the multivariable model. Finally, a multivariable logistic regression model was fitted to identify variables associated with knowledge of LAM. Adjusted odds ratios (AORs) with a 95% confidence interval were used to display the association between the outcome and the predictors. The significance level of 0.05 was used as a cut-off point for all statistical tests.

### Ethical considerations

Ethical clearance for this study was obtained from the Institutional Health Research Ethics Review Committee of the University of Gondar (Ref No. O/V/P/RCS/05/3073/2017). The study participants gave informed consent, and confidentiality of the obtained information was maintained. All methods were carried out in accordance with protocol guidelines and ethical regulations.

## Results

### Socio-demographic characteristics

Of the 3319 participants approached, 3148 (94.8%) were included in the study, while 5.2% of them refused to respond and discontinued before finishing the response. The majority of the study participants were 21–30 years old (71.7%), urban residents (92.1%), and gave birth vaginally (78%). Details of the sociodemographic characteristics of the participants are indicated in Table 1.

**Table 1:** Basic characteristics of study participants of knowledge about LAM in Ethiopia.

Variables	Frequency	Percent
Age		
15–20	518	16.4
21–25	1164	37.0
26–30	1092	34.7
31–35	258	8.2
36–40	116	3.7
Total (n)	3148	100
Place of residence		
Rural	249	7.9
Urban	2899	92.1
Total (n)	3148	100
Maternal education		
No formal education	794	25.2
Read and write	126	4.0
Primary	662	21.1
Secondary	860	27.3
College and above	706	22.4
Total (n)	3148	100
Study site		
Harar	523	16.6
Addis Ababa	495	15.7
Jimma	527	16.7
Jigjiga	545	17.3
Gondar	549	17.5
Hawasa	509	16.2
Total (n)	3148	100
Partner’s occupation		
Farmer	322	10.5
Government employee	1134	36.8
Private	443	14.4
Merchant	455	14.8
Other	724	23.5
Total (n)	3078	100
Place of delivery		
Health center	72	2.3
Hospital	3076	97.7
Total (n)	3148	100
Mode of delivery		
Cesarean section	690	21.9
Vaginal	2458	78.1
Total (n)	3148	100
Received FP counseling during ANC		
No	522	28.6
Yes	1302	71.4
Total (n)	1824	100
Heard of LAM		
No	936	32.6
Yes	1940	67.4
Total (n)	2876	100
Ever used LAM		
No	1285	67.5
Yes	619	32.5
Total (n)	1904	100

*LAM* lactation amenorrhea method, *FP* family planning, *ANC* antenatal care.

### Knowledge of LAM and its associated factors

Of the 3148 postpartum women surveyed, 42.3%, 71.6%, and 72.9% correctly identified the length and duration of its protection, menses conditions, and complementary feeding, respectively. Overall, 40.6% 95 CI (38.9, 42.3) of the women were knowledgeable about LAM (Table 2).

**Table 2:** Knowledge of LAM among postpartum women in Ethiopia (n = 3148).

Variable	Frequency	Percent
How long does it prevent (months)?		
** < **6 months^a^	1330	42.3
> 6 months	1818	57.7
Complementary food is not allowed		
Yes^a^	2254	71.6
No	894	28.4
The woman should be amenorrheic		
Yes^a^	2294	72.9
No	854	27.1
Comprehensive knowledge about LAM		
Yes^a^	1278	40.6
No	1870	59.4

^a^Indicates the correct response.

After binary logistic regression, variables that had *p* < 0.25 includes: educational level, maternal occupation, place of delivery, fertility desire, parity, distance from a health facility, and receiving counselling about LAM. These variables were analyzed using multivariable logistic regression.

Knowledge of LAM was associated with educational status, parity, distance from health facilities, fertility desire, receiving counseling about LAM, and place of delivery (Table 3). Participants who attended a college education or above were 2.1 (AOR = 2.1; 95% CI 1.5–2.8) times more likely to have knowledge of LAM compared to those who do not have a formal education. Compared to primiparous, those who gave birth to two and three or more children were 2.3 (AOR = 2.3; 95% CI 1.6–3.6) and 2.4 (AOR = 2.4; 95% CI 1.5–4.0) times more likely to have knowledge of LAM, respectively. Participants who live more than 30 min from the nearest health facility were 30% (AOR = 0.70; 95% CI 0.6–0.8) less likely to have knowledge of LAM compared to those living close to health facilities. Moreover, a participant who reported a desire to become pregnant within the coming 2 years were 1.3 (AOR = 1.3, 95% CI 1.1, 1.5) times more likely to have knowledge of LAM. Participants who reported getting counseling about LAM were 3.0 (AOR = 3.0; 95% CI 2.6–3.7) times more likely to be knowledgeable about LAM compared to their counterparts. Finally, participants who gave birth in a hospital were 2.6 (AOR = 2.6; 95% CI 1.4–2.6) times more likely to have more knowledge of LAM compared to those who gave birth in a health center.

**Table 3:** Factors associated with knowledge of LAM among postpartum mothers in Ethiopia (n = 3148).

Variables	Categories	aOR (95% CI)	*p* value
Education level	No formal education	Reference	
	Read and write	0.8 (0.7, 1.3)	0.41
	Primary	0.9 (0.7, 1.1)	0.27
	Secondary	0.8 (0.7, 1.1)	0.3
	College and above	2.1 (1.5, 2.8)	< 0.01
Maternal occupation	Farmer	Reference	
	House wife	0.9 (0.5, 1.7)	0.71
	Government employee	0.8 (0.4, 1.8)	0.80
	Private	0.4 (0.2, 1.1)	0.13
	Merchant	1.2 (0.62, 2.5)	0.50
	Other	0.6 (0.3, 1.3)	0.16
Place of delivery	Health center	Reference	
	Hospital	2.6 (1.4, 2.6)	< 0.01
Fertility desire	No	1	
	Yes	1.3 (1.1, 1.5)	< 0.01
Parity	Para 1	Reference	
	Para 2	2.3 (1.6, 3.6)	< 0.01
	Para 3 and above	2.4 (1.5, 4.0)	< 0.01
Distance from health facility (how long it takes to drive by car)	< 30 min	Reference	
	≥ 30 min	0.70 (0.6, 0.84)	< 0.01
Received counseling on LAM	No	Reference	
	Yes	3.0 (2.6, 3.7)	< 0.01

*aOR* adjusted odds ratio.

## Discussion

This study was conducted to assess knowledge of LAM among postpartum mothers in Ethiopia. The proportion of partipants who were knowledgeable about LAM was 40.6% (95% CI 38.9–42.3). Educational level, parity, distance from a health facility, fertility desire, getting counseling about LAM, and place of delivery were factors associated with knowledge of LAM.

The proportion of knowledgeable participants about LAM in this study is higher than those in previous studies from Kochaeli-Turkey which was 25.68%^23^, Aksum (Ethiopia) which was 8.8%^21^, and 28.6% in a district in eastern Turkey^9^. This variation in proportion could be due to differences in the characteristics of the study population, design, time, and settings. For instance, the study in Turkey used a prospective randomized study design among all women of reproductive age, while our study included postpartum mothers who gave birth within the last 7 days. In addition, the Aksum study used a community-based design among mothers 1 year after giving birth, while the one from eastern Turkey was community-based too. Our finding is, however, lower than studies from Uganda (56%)^10^ and Indonesia (59.6%)^4^. This difference might be related to the data source, study population, or study design. The Ugandan study included only 15–24-year-old women, compared to our inclusion of all postpartum mothers.

Consistent with previous reports, participants with college and above education were more likely to have knowledge of LAM compared to those with no formal education^10,21,23^. A literate participant might have a better chance of seeking and accessing more information and awareness about contraception. Similarly, knowledge about LAM was found to be higher among multiparous compared to primiparous mothers. Participants with prior birth experience will have an opportunity for counseling on LAM or other contraceptives in general. The association of counseling by health extension workers and postnatal care follow-up visits with knowledge of LAM was previously reported^21^.

Participants who reported a desire to have a child within 2 years were more knowledgeable compared to their counterparts. This might be related to the mother’s discussion about short-term contraceptive options with fewer side effects and an immediate pregnancy return. Such mothers might get additional information from the mass media or their social networks^7^. By the same token, we found mothers who gave birth in hospitals were more likely to be knowledgeable compared to those who gave birth in health centers. This might be related to the difference between midwives and other care providers working at hospitals and health centers in terms of professional experiences, which may affect the quality of information they provide to mothers during counseling. Training midwives in contraceptive counseling would ensure mothers receive accurate information about available options^24^.

As expected, women living away from health facilities were less likely to have knowledge of LAM compared to those living within a 30-min radius of driving by car. Given that participants living far from facilities have limited access to health facilities, including counseling and exposure, this finding was not unexpected. The positive association between proximity to health facilities and contraceptive choices was documented in Ethiopia^25^. Our study is a multicenter study comprising randomly selected regional states and city administrations, making our findings generalizable to Ethiopia.

## Conclusion

In this study, we found that the proportion of postpartum women who were knowledgeable about LAM was relatively low. Improving women’s education, strengthening counseling on LAM, as a means of contraception in ANC services and with health extension services, and improving health facility services are essential for enhancing knowledge of lactation amenorrhea as a method of contraception.

### Strengths and weaknesses of this study

The strength of this study is that it is a multicenter study, including the eastern, western, northern, southern, and central parts of the country, as well as involving different sociodemographic statuses of the Ethiopian postpartum mothers. The fact that it is an interviewer-administered questionnaire is also one of the strengths of the study for participants who can’t read and write, though it can cause interviewer bias, which is the weakness.

## Data Availability

As it is mentioned in the Methodology part, this study is part of a larger multi-center national study on the effectiveness of LAM in Ethiopia, and the raw data contains other components of the larger study and person-identifying data that we can’t share publicly. However, all the data generated or analyzed for this objective is included in this article, and non-person-identifying data can also be accessed from the corresponding author upon reasonable request.

## Abbreviations

LAM: Lactational amenorrhea method
SPMMCH: Saint Paul Millennium Medical College Hospital
SNNPR: Southern Nations, Nationalities, and Peoples’ Region
ODK: Open data kit
HCG: Human chorionic gonadotropin
